# Immunodominance of Antigenic Site B over Site A of Hemagglutinin of Recent H3N2 Influenza Viruses

**DOI:** 10.1371/journal.pone.0041895

**Published:** 2012-07-25

**Authors:** Lyubov Popova, Kenneth Smith, Ann H. West, Patrick C. Wilson, Judith A. James, Linda F. Thompson, Gillian M. Air

**Affiliations:** 1 Department of Biochemistry & Molecular Biology, University of Oklahoma Health Sciences Center, Oklahoma City, Oklahoma, United States of America; 2 Department of Chemistry and Biochemistry, University of Oklahoma, Norman, Oklahoma, United States of America; 3 Arthritis and Clinical Immunology Research Program, Oklahoma Medical Research Foundation, Oklahoma City, Oklahoma, United States of America; 4 Immunobiology and Cancer Program, Oklahoma Medical Research Foundation, Oklahoma City, Oklahoma, United States of America; 5 Section of Rheumatology, Department of Medicine and Committee on Immunology, University of Chicago, Chicago, Illinois, United States of America; Fudan University, China

## Abstract

H3N2 influenza viruses have now circulated in the human population for 43 years since the pandemic of 1968, accumulating sequence changes in the hemagglutinin (HA) and neuraminidase (NA) that are believed to be predominantly due to selection for escape from antibodies. Examination of mutations that persist and accumulate led to identification of antigenically significant mutations that are contained in five antigenic sites (A–E) mapped on to the H3 HA. In early H3N2 isolates, antigenic site A appeared to be dominant while in the 1990s site B seemed more important. To obtain experimental evidence for dominance of antigenic sites on modern H3 HAs, we have measured antibodies in plasma of human subjects who received the 2006–07 trivalent subunit influenza vaccine (H3 component A/Wisconsin/67/05) or the 2008–09 formulation (H3 component A/Uruguay/716/07). Plasmas were tested against expressed HA of Wisconsin-like influenza A/Oklahoma/309/06 and site-directed mutants in antigenic site A (NNES121-124ITEG, N126T, N133D, TSSS135-138GSNA, K140I, RSNNS142-146PGSG), and antigenic site B (HL156-157KS, KFK158-160GST, NDQI189-192QEQT, A196V). “Native ELISA” analysis and escape mutant selection with two human monoclonal antibodies demonstrated that antibody E05 binds to antigenic site A and 1_C02 binds to site B. We find that most individuals, after vaccination in seasons 2006–07 and/or 2008–09, showed dominance of antigenic site B recognition over antigenic site A. A minority showed dominance of site A in 2006 but these were reduced in 2008 when the vaccine virus had a site A mutation. A better understanding of immunodominance may allow prediction of future antigenic drift and assist in vaccine strain selection.

## Introduction

Influenza viruses are major pathogens that cause seasonal epidemics and global pandemics. Each year in the United States more than 200,000 people are hospitalized and 20,000–36,000 people die from flu-related complications [1]. Due to rapid accumulation of mutations to escape host defense mechanisms, the vaccine components must be frequently updated to protect the human population against influenza. There are three types of influenza viruses, A, B and C. Type A viruses are divided into subtypes according to cross-reactivity of sera with viral surface glycoprotein antigens; to date these are subtypes H1 to H16 of the hemagglutinin (HA) and N1 to N9 of neuraminidase (NA) although an H17 has been recently proposed [2]. H1N1 and H3N2 along with type B viruses are currently circulating in the human population and these are the antigens in the trivalent vaccines. HA is involved in two steps of the process of influenza infection. It binds the virus to sialic acid residues of glycoproteins or perhaps glycolipids that act as receptors on host cells then, following endocytosis, HA mediates the fusion of viral and cellular membranes to allow release of the viral genome-polymerase complex into the cell (reviewed by Skehel and Wiley [3]). Neutralizing antibodies directed against the hemagglutinin are considered the most protective against influenza virus infection and vaccine responses are most commonly tested by hemagglutination-inhibition assays.

**Figure 1:**
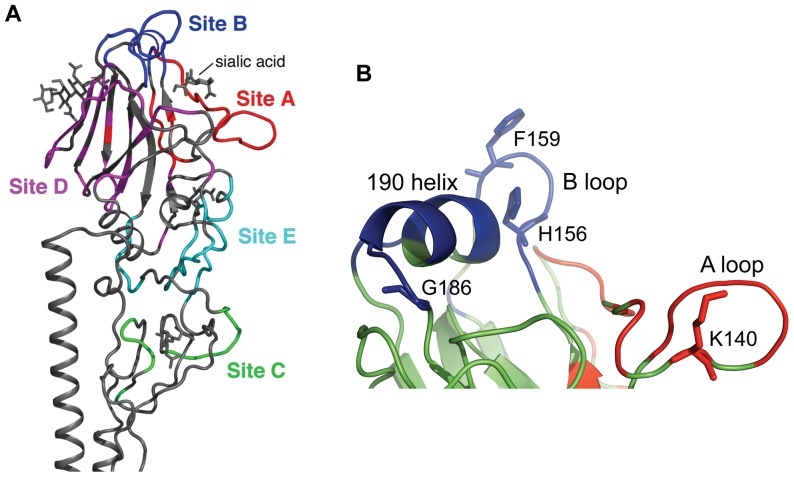
Antigenic structure of H3 HA. Five antigenic sites A–E are mapped on the HA1 surface of H3N2 influenza viruses. (**A**). Antigenic site A (red color) and antigenic site B (blue color) are localized on the top of HA around the receptor binding pocket. (**B**). The “190 helix” and “B loop” create antigenic site B. The “A loop” is a part of antigenic site A. Figure 1A was made from PDB ID 2VIR [24] using PyMol (Schrödinger, LLC). Figure 1B was made from an Oklahoma/309 HA structural model made by SWISS-MODEL.

To escape from neutralizing antibodies produced in response to infection and, most recently, mass vaccination, changes in HA have accumulated in a process named antigenic drift over the 43 years since the H3N2 subtype of influenza virus was first isolated from humans in 1968. From 1968 to 2010 there have been 108 amino acid changes identified at 63 residue positions in HA1 (total length 328 amino acids) in the major epidemic strains and most of these changes are considered to result from antigenic drift because the majority (85.5%) are clustered into regions called antigenic sites. “Antigenic site” was an operational term introduced by Gerhard and Webster [4] to describe specificities of monoclonal antibodies (mAbs). Antibodies that competed with each other for binding were considered to bind the same antigenic site. Webster and Laver identified four antigenic sites on the surface of H3 HA (A–D) by competition assays [5] and Skehel identified a fifth antigenic site, E [6]. Each antigenic site contains many epitopes, structurally defined as the amino acids on the antigen that contact amino acids of the antibody [7]. Competition between antibodies that bind the same site suggested that epitopes in the same site are physically overlapping but are distinct, and no one antibody molecule binds to the whole of an antigenic site. Evidence for the location of epitopes came from characterization of escape mutants, selected by mAbs, that contain single amino acid substitutions that reduce binding of the mAb to undetectable levels [6], [8]–[11]. The three-dimensional structure of A/Aichi/2/68 X-31 HA [12] showed the location of escape mutations selected by monoclonal antibodies. Assuming that the amino acid that changes in an escape mutant is within the epitope, there was now an indication of where the antigenic sites are located. Wiley and Wilson [9], [13] took into account the sites of all known escape mutations and their corresponding antigenic site assignment, together with changes in naturally circulating viruses from 1968 to the mid-1980s, to suggest the physical boundaries of sites A–E on H3 HA, and they compiled a directory of amino acids in each of antigenic sites A–E (Figure 1A) [9]. This is the map we and others have been using but it important to stress that (i) most aspects of this map were not experimentally confirmed and (ii) we do not know if this map, which was developed based on 1968 and 1971 isolates, applies to currently circulating viruses. Certainly many of the amino acids on the Wiley and Wilson list appear to be important in antigenic drift when studied phylogenetically [14] or experimentally [15].

Some mutations in HA1 created new sites for attachment of oligosaccharide chains and since the beginning of the Hong-Kong pandemic period in 1968 the number of N-linked glycan attachment sites in HA1 has increased from three to eleven. Accumulation of oligosaccharide chains in antigenic sites has been suggested to contribute to immune evasion [6], [16]–[18].

Antibodies directed against the head of the HA are the most abundant of the neutralizing antibodies after vaccination. X-ray structures of complexes of mAb Fabs bound to HA show how antibodies can block binding of the viral HA to sialic acid receptors on host cells [19]–[21]. Antibodies were shown to bind near the sialic acid binding site or somewhat distant but in an orientation where the Fc portion would obstruct binding to cellular receptors [19], [22]–[24]. Monoclonal antibodies specific to the more conserved stem domain of H1N1, H3N2 and H5N1 viruses have been described in recent studies [25]–[29]. These antibodies have broad neutralizing activity between viral subtypes and apparently act by blocking the conformational change that leads to fusion [27]. The broadly neutralizing antibodies that bind to the stem region have not been detected as a significant component of the antibody repertoire but they were found to be induced by a “headless” construct [30]. At this time vaccine production is still re-tooled annually according to new antigenic variants that are altered in the traditional neutralizing antigenic sites in the receptor binding domain of HA1.

Antibodies with flat binding surfaces cannot penetrate the receptor binding site of HA [31] but they can sterically block receptor attachment by binding to epitopes on the loops surrounding the sialic acid binding site [12], [13]. The problem is that mutations in these loops can abrogate antibody binding without affecting HA function. One crystal structure shows an anti-H1 antibody in which the CDR3 loop is long enough to enter the receptor binding site, giving a degree of cross-reactive neutralization among seasonal H1N1 viruses [21], but in most cases the virus can easily escape antibody neutralization.

The presence of five independent antigenic sites on HA would appear to mean that at least 5 amino acid sequence changes would be needed for a new antigenic variant to emerge. In the early years of H3N2 circulation, this appeared to be the case [13] but in recent years the changes that necessitate a change in vaccine strain have been fewer in number. The host immune response may be limited to only the most immunodominant antigenic sites of HA.

Early studies of antigenic sites on HA of A/Memphis/1/71 suggested that antigenic site A was immunodominant [32]. In this study only variants changed in antigenic site A were discriminated by polyclonal antiserum. However, a rabbit was immunized with a mAb-selected escape mutant of A/Memphis1/71 that had the change G144D in HA1 and the serum was absorbed with wild type virus so that only antibodies against the new epitope would remain. There were not any; the changed epitope was not immunogenic, indicating a change in immunodominance. Studies by Temoltzin-Palacios and Thomas [33], [34] showed that the neutralizing Ab response of CBA/Ca mice is focused on a few regions of the HA1 subunit after intranasal infection with A/Aichi/2/68 X-31 virus. Sequence analyses of variant viruses isolated after a second infection of mice showed that 60% of analyzed viral HAs had a G158E mutation and 17% contained a D61N mutation. It was concluded that antigenic sites B and E are immunodominant in mice infected with the X-31 virus. Six polyclonal human plasma samples collected in 1976 with hemagglutinin-inhibition activity against Aichi/68 showed decreased binding to mutants in antigenic site A [35] which correlates with the data obtained with mouse mAbs specific to site A discussed above. A computational study suggested that site A was immunodominant in 1968–1971 and 1989–1995 while site B was dominant in 1972–1987 and 1996–2003 [36]. Studies with human sera have given mixed results of clear immunodominance of site A in 1991 [37] and part of site B in 1998–99 sera [15] but no clear dominance was seen in a study of sera collected in 2004 [38].

Overall, the phylogenetic analyses [14], [39] and serum studies [15], [37] suggest that sites A and B are the most important in directing antigenic drift of H3N2 human viruses, and so we have investigated the immunogenicity of antigenic sites A and B of recent H3 HAs. We mapped the binding of two human monoclonal antibodies to wild type A/Oklahoma/309 HA and mutant HAs derived from it, and we tested the reactivity of polyclonal antibodies in human plasma samples after seasonal vaccination in 2006 (H3N2 2006–07 component A/Wisconsin/67/05) and/or after vaccination in 2008 (H3N2 2008–09 component A/Uruguay/716/2007), to wild type HA and mutants in antigenic sites A and B. Our results indicate that most neutralizing antibodies in human plasma against both vaccine strains A/Wisconsin/67/05 and A/Uruguay/716/07 are directed to antigenic site B. We conclude that antigenic site B is immunodominant over site A in recently circulating H3N2 viruses and that site B mutations may drive the next antigenic drift.

## Results

### Design of mutants

For this study we made mutations in antigenic sites A and B in HA1. To test if mutations accumulated since 1968 have changed the map of antigenic sites on HA and, second, to map epitopes of monoclonal antibodies made against a recent virus, we mutated the sequence of HA1 of a local Wisconsin-like virus, A/Oklahoma/309/2006 (H3N2), to those amino acids in HA1 of the earliest human H3N2 virus A/Aichi/2/1968. The mutations are summarized in Table 1.

**Table 1:** Mutations made in antigenic site A and B of A/Oklahoma/309/2006 HA.

Mutations made^1^	Change	Site	Charge change	Other
NNES121-124ITEG	N121I	A	0	
	N122T	A	0	Remove glycosylation site
	S124G	A	0	Remove side chain
N126T	N126T	A	0	Remove glycosylation site
N133D	N133D	A	1-	Remove glycosylation site
TSSS135-138GSNA	T135G	A	0	Remove side chain
	S137N	A	0	
	S138A	A	0	
K140I	K140I	A	1-	Same position as E05 escape mutant
RSNNS142-146GPGSG	R142G	A	1-	Remove side chain
	S143P	A	0	
	N144S	A	0	Remove glycosylation site
	S146G	A	0	Remove side chain
				
HL156-157KS	H156K	B	1-	Remove stacking with F159
	L157S	B	0	
KFK158-160GST	K158G	B	1-	Remove side chain
	F159S	B	0	Same as1_C02 escape mutant
	K160I	B	1-	
NDQI189-192QEQT	N186Q	B	0	
	D187E	B	0	
	I190T	B	0	
A196V	A196V	B	0	

1 The amino acids were changed to those of HA of A/Aichi/68.

### Expression of HA in the Bac-to-Bac® expression system

Initially we expressed HA in a full-length form in mammalian cells, but the level of HA on the cell surface was too low to reliably quantify antibody binding. We therefore used a Baculovirus expression system with a synthetic codon-optimized gene that deleted the transmembrane domain and included a trimerization sequence [40]. HA expression from the synthetic gene was greater than 100 fold more than full-length HA expressed in mammalian cells, and >90% of baculovirus-expressed HA was secreted into the supernatant.

**Figure 2:**
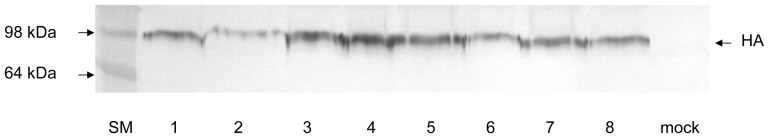
Expression of wild type and mutant HAs in the baculovirus system. 1. Wild type 309 HA 2. Mutant HL156-157KS 3. Mutant KFK158-160GST 4. NDQI189-192QEQT 5. A196V 6. N133D 7. TSSS135-138GSNA 8. K140I. Supernatant (25 µl) from a 25 cm^2^ flask of Sf9 cells infected with the recombinant baculoviruses expressing wild type and mutant HA was loaded on a 12% SDS-polyacrylamide gel. HA was visualized by immunoblotting assay with anti-HA tag (YPYDVPDYA) polyclonal antiserum.

**Figure 3:**
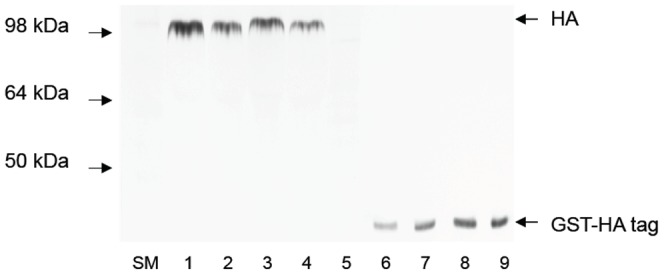
Quantitation of expressed HA by immunoblotting assay using anti-HA-tag antibody. Different concentrations of standard protein GST-HA tag were visualized in the same membrane as HAs. 1–2, 309 HA (10 µl, 7 µl); 3–4, mutant HA HL156-157KS (10 µl, 7 µl); 5, mock infected; 6–9. GST-HA tag 4 ng, 6 ng, 8 ng, 10 ng, respectively. The bands were scanned and quantitated using ImageQuant software (Molecular Dynamics). Amounts of HA were determined from a standard curve of GST-HA tag fusion protein. Standard curves were built for each sample of HA.

Based on this we made the HA mutants (Table 1) in the baculovirus system. All the mutants were expressed (Figure 2). We quantitated wild type and mutant HAs by Western blot analysis using a commercial anti-HA tag antibody taking advantage of the HA-tag sequence (YPYDVPDYA) that is conserved in all H3 HAs (Figure 3).

We confirmed the correct folding of wild type and mutant HAs expressed from baculovirus using trypsin digestion; trypsin cleaved the expressed HA0 into HA1 and HA2 while misfolded HA would have been degraded into small peptides [41], [42].

### Effect of mutations in HA of A/OK/309/06 on binding to human monoclonal antibodies

We measured the affinity of binding of purified human monoclonal antibodies (hmAbs) 1_C02 (D1–7) and E05 (D3–4) [43] to wild type and mutant HAs using a native ELISA, capturing the HA by its His6-tag to avoid denaturation (Table 2). The V_H_ and V_L_ genes for hmAb E05 and 1_C02 were from single B cells of patients vaccinated with trivalent subunit vaccine containing H3N2 components A/California/7/04 and A/Wisconsin/67/05 X-161b) respectively. HmAbs 1_C02 and E05 bind intact virions of A/Wisconsin/67/05 X-161b and Wisconsin-like isolate A/Oklahoma/309/06 with high affinity but show little or no binding to related H3N2 viruses when tested in a native ELISA (Table 3). Our ELISA results with recombinant mutant HAs show that the binding site of 1_C02 is site B. We found that mutations in antigenic site B decrease the affinity of binding of mAb 1_C02 to HA from a Kd of 1 nM in wild type to 6 nM in mutant KFK158-160GST while there was no detectable binding to HL156-157KS. There was no significant change in binding to NDQI186-190QEQT and A196V. No mutations in antigenic site A affect the binding of 1_C02. We found that E05 binds to site A. Mutations K140I and RSNNS142-146GPGSG in antigenic site A abolished the binding to E05 but other mutations in antigenic site A NNES121-124ITEG, N126T, N133D and TSSS135-138GSNA had no effect. No mutations in antigenic site B affected the binding of E05. Our results (Table 2) show that the epitopes of human mAbs E05 and 1_C02 are contained in antigenic sites A and B respectively (Figure 1B). It is important to emphasize that these epitopes do not encompass the whole of each antigenic site, which would be physically impossible given the size of an antibody footprint compared to the large surface areas assigned to sites A or B.

**Table 2:** Binding of human monoclonal Abs 1_C02 and E05 to 309 HA and mutants.

HA1^1^	Antigenic site	Kd, nM ± SD	
		1_C02	E05
309 wt	Wild type	1.0±0.2	2.0±0.1
NNES121-124ITEG	A	2.1±0.3	1.2±0.1
N126T		1.8±0.3	1.1±0.1
N133D		0.8±0.2	0.6±0.3
TSSS135-138GSNA		1.0±0.3	0.6±0.2
K140I		1.0±0.4	>1000
RSNNS142-146GPGSG		1.3±0.4	>1000
HL156-157KS	B	>1000	0.3±0.1
KFK158-160GST		6.0±3	0.8±0.2
NDQI189-192QEQT		1.5±0.3	1.0±0.3
A196V		1.1±0.2	1.9±0.1

1 His_6_-tagged wildtype and mutant HA was expressed in insect cells and purified on a nickel column.

**Table 3:** Dissociation constants (Kd) for binding of human monoclonal Abs 1_C02 and E05 to H3N2 viruses.

	Kd (nM) ± SD	
	1_C02	E05
A/Beijing/89	No binding	No binding
A/Panama/99	No binding	No binding
A/Wyoming/03	>1000	No binding
A/California/04	No binding	97.8±26.3
A/Wisconsin/05	2.1±0.3	6.6±0.5
A/Oklahoma/06	1.0±0.2	2.0±0.3
A/Uruguay/07	No binding	No binding
A/Perth/09	No binding	No binding
EM 1_C02 (F159S)	No binding	6.0±0.5
EM E05 (K140E/T)	1.9±0.1	No binding

### Selection of escape mutants with human mAbs

We selected escape mutant viruses after growth of A/Wisconsin/67/05-X161b virus in the presence of antibody 1_C02 or E05. An escape mutant virus selected by 1_C02 (EM1_C02) contains a substitution F159S in antigenic site B of HA1. Two escape mutants selected by E05 had amino acid substitutions at 140 (K140E and K140T) in antigenic site A. The binding constants (Kd) are included in Table. 3. These results confirm the binding of hmAb E05 to an epitope within site A and of 1_C02 to an epitope within site B.

### Reactivity of wild type and mutant HAs with polyclonal antibodies in human plasma after vaccination

**Figure 4:**
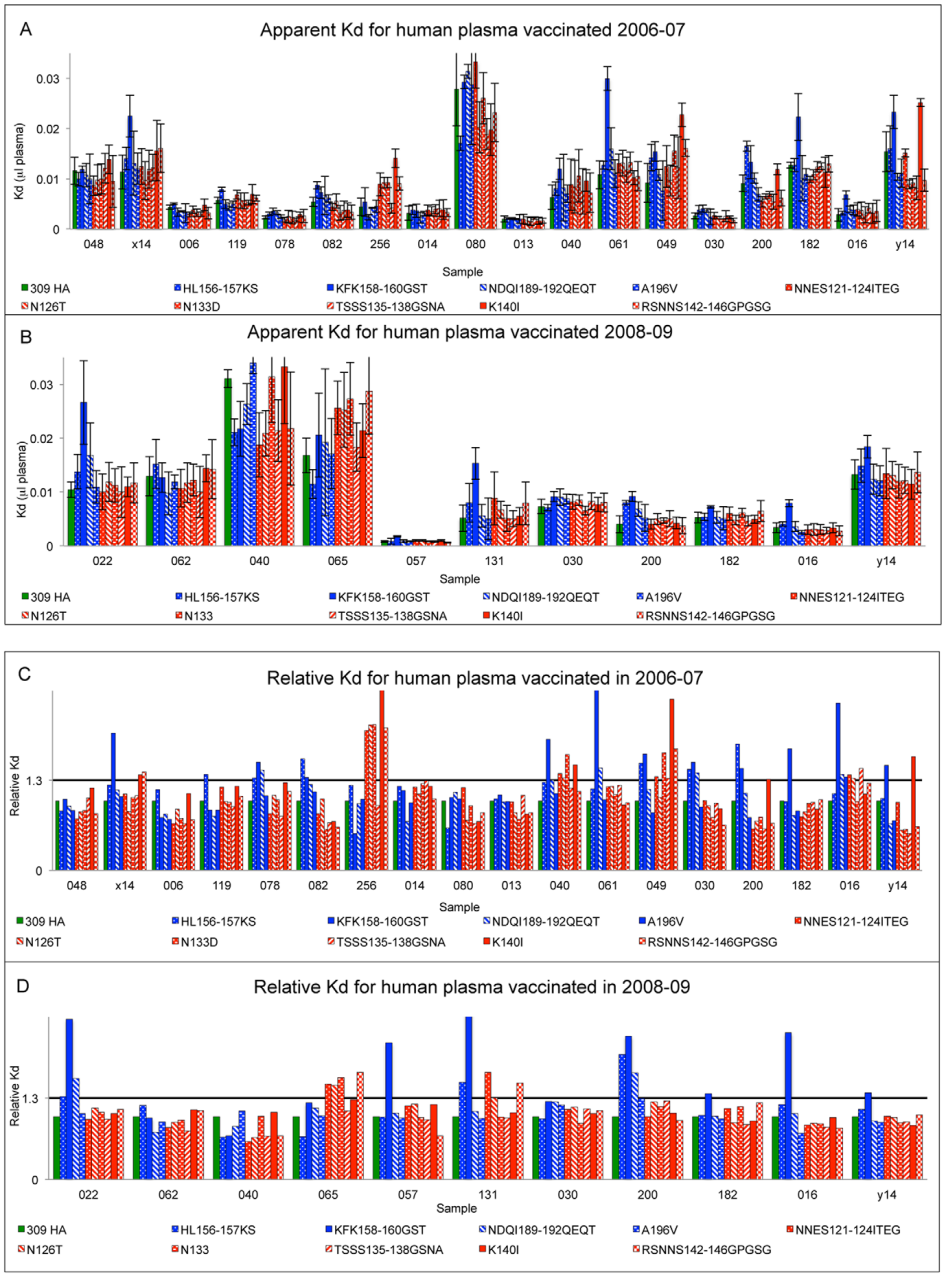
Analysis of human plasma samples vaccinated in season 2006–07 (H3 component A/Wisconsin/67/05) and season 2008–09 (H3 component A/Uruguay/716/07). **A**, **B**. Overall affinity (Kd ± St. dev.) of antibodies in human plasma against wt 309 HA and mutants. Overall affinity labeled as dissociation constant (Kd) of binding of antibodies in plasma from subjects to wild type and mutants in antigenic site A or B; higher Kd is lower affinity. Kds were measured as described in Materials and Methods and the units are µl plasma in the standard assay. Results are plotted as mean Kd ± standard deviation over 3 experiments. **C**, **D**. Relative Kd of antibodies in human plasma against mutants relative to binding of wild type A/OK/309/06 HA. Kds of binding of plasma antibodies to mutants HA are calculated by normalizing Kd of wild type 309 HA to 1.0.

To test antibodies in human plasma after vaccination we used the same panel of mutants. If antibodies in human plasma are dominantly expressed against epitopes in antigenic site A or B we will observe reduced binding of sera to mutants in this antigenic site. We used 18 human plasma samples collected 6 weeks after vaccination in Fall 2006 (H3 component A/Wisconsin/67/05) and 11 vaccinated in Fall 2008 (H3 component A/Uruguay/716/07). We tested binding of plasma antibodies to wild type and mutant baculovirus-expressed HA, captured on His-tag antibody plates to ensure preservation of native structure. The overall avidity of binding (Kd_apparent_) of plasma antibodies after vaccination in 2006 or 2008 are shown in Figure 4 and Tables S1 and S2. There is a wide range of avidities among the different plasmas (Figure 4, panels A and B) and so we normalized the results relative to wildtype and looked for significantly reduced binding (Kd increased ≥30%) to mutants in site A or site B or both (Figure 4, panels C and D).

The results are summarized in Table 4. Twelve of 18 subjects vaccinated in 2006 showed reduced binding to site B mutants; 11 of these 12 showed significantly reduced affinity to the mutant KFK158-160GST. Only 7 of 18 subjects showed reduced binding to site A mutants; 6 recognizing the mutation K140I. After vaccination in 2008, 7 of 11 subjects discriminate site B mutants, and all 7 recognized the KFK158-160GST mutant. Only 2 of the 11 showed reduced binding to site A mutants. Six subjects were vaccinated in both seasons. All 6 showed reduced binding to site B mutants in 2006 and 4 of 6 in 2008. Four of the six showed reduced binding to site A mutants in 2006 but none in 2008.

**Table 4:** Decreased binding to site A and B mutants by human plasma after vaccination.

Mutant	Vaccinated 2006–07 (18 subjects)		Vaccinated 2008–09 (11 subjects)	
	Number^1^	Percent	Number	Percent
**Site B**				
HL156-157KS	6	33	3	27
KFK158-160GST	11	61	7	64
NDQ189-192QEQT	5	28	2	18
A196V	1	0	0	0
Total subjects binding site B	12	66	7	64
**Site A**				
NNES121-124ITEG	4	22	2	18
N126T	3	17	2	18
N133D	3	17	1	9
TSSS133-138GSNA	2	11	0	0
K140I	6	33	0	0
RSNNS142-146GPGSG	3	17	2	18
Total subjects binding site A	7	39	2	18
Total A, B or both	13	72	8	73
Not A or B	5	28	3	27

1 Number of subjects who showed decreased avidity (Kd increased by ≥30%) to the mutant HA.

### Human plasma antibodies after vaccination against A/Wisconsin/67/05 or A/Uruguay/716/07 have low binding to escape mutant virus EM 1_C02

**Figure 5:**
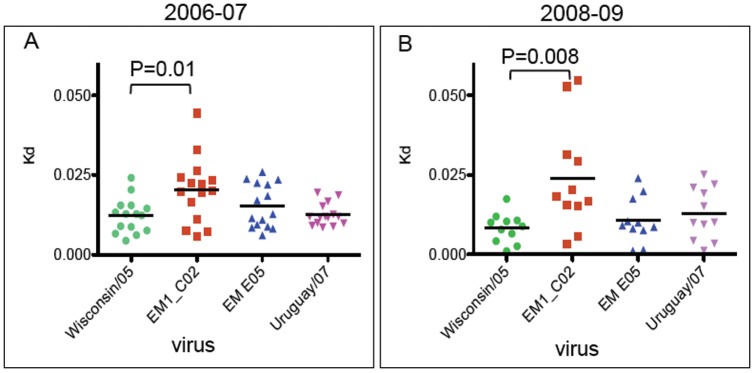
Overall dissociation constants (Kd) of antibodies in human plasma after vaccination. Kd of antibodies in season 2006–2007 (A) and season 2008–2009 (B). Plasma samples were tested against H3 vaccine viruses A/Wisconsin/67/05 (2006–2007 vaccine) and A/Uruguay/716/07 (2008–2009) and against escape mutant viruses EM 1_C02, EM E05. The median Kd is represented by a horizontal bar, and the p values are from the Student's T test.

We measured binding of post-vaccination plasma samples (15 from 2006 and 11 from 2008 vaccinations) to A/Wisconsin/67/05 virus and escape mutants derived from it using the native ELISA technique, this time capturing virus by binding to sialylated glycans on turkey erythrocyte ghosts used to coat the wells. Only virions with native HA will attach to the sialic acids. A/Wisconsin/67/05 and A/Uruguay/716/07 viruses and escape mutants derived from Wisconsin/05 virus were titrated with plasma dilutions to generate binding curves and estimate the overall apparent affinity (Kd_apparent_). Our results show that on average antibodies in human plasma samples in both seasons have reduced binding to escape mutant virus EM1_C02 but not to EM E05 (Figure 5). These data are in accord with the results of testing human plasma antibodies against mutant HAs (Figure 4) in that antibodies in the majority of human plasmas bind to epitopes within antigenic site B and only a minority to antigenic site A after vaccination in 2006 and even fewer in 2008.

## Discussion

In the early years after the emergence of H3N2 viruses in humans multiple sequence changes were observed from one epidemic isolate to next. During the first 10 years (1968–1979), 33 amino acid changes accumulated in HA1 (10.1%) but the vaccine was changed only 3 times. In recent years the vaccine has been changed more frequently even though there are fewer changes in circulating viruses [44]. For example, the only consistent differences between Wisconsin-like viruses and Brisbane/Uruguay-like viruses are G50E in site E and K140I in site A (Table 5). These two changes were sufficient for the H3N2 component of vaccine to be changed for season 2008–09.

**Table 5:** Differences in HA1 sequence between Wisconsin/05 and Uruguay/07 PR8 reassortant vaccine strains.

Amino acid	**50**	122	**140**	**142**	**156**	186	**188**	**194**	**196**	223
Site	**C**	-	**A**	**A**	**B**	-	**B**	**B**	**B**	-
California/7/04	**G**	(N)^1^	K	R	H	G	N	L	T	V
Wisconsin/67/2005 X-161b	**G**	(D)^2^	K	R	(Q)^3^	(V)^4^	D	L	A	(I)^5^
Oklahoma/309/2006	**E**	N	K	R	H	G	D	L	A	V
Brisbane/10/07	**E**	N	**I**	R	H	G	D	(P)^6^	A	V
Uruguay/716/2007 X-175	**E**	N	**I**	(G)^7^	H	G	D	(P)^6^	A	V

1 Changes in parentheses are not present in other isolates from the same season, so may not be antigenically significant. The percent variation analyses were done using the Influenza Research Database using the “Analyze Sequence Variation” [52].

2 All Wisconsin/67/05 sequences have D but 99.7% of 2005/6 isolates have N.

3 Q in PR8 reassortants, not isolates from primary chick kidney cells or egg passages.

4 V in PR8 ressortants and some other Wisc/67/05 entries; 98.8% of 2005–6 isolates have G.

5 I in all Wisc/67/05 entries but 97% of 2005/6 isolates have V.

6 P in egg-passaged viruses but not many others.

7 G only in reassortant X-175.

### Antigenic map of H3 HA

Wiley and Skehel proposed a list of amino acids contributing to each of the five antigenic sites A–E (Figure 1A) [9], but most of these have not been experimentally tested. The PDB database contains 4 crystal structures of mAbs bound to X-31 HA (PDB IDs 2VIR, 1QFU, 1E08, 1KEN) and while these confirm the locations of immunogenic regions, there is some overlap of the classical 5 sites; for example, antibody HC19 contacts 5 amino acids assigned to Site A as well as 5 listed in Site B [19], [20]. As antigenic drift proceeded from 1968 to 2011, the amino acid sequence changes are clustered, but not exclusively, into the proposed antigenic sites, but there are no structural maps of neutralizing epitopes on the receptor binding domain of newer H3 viruses. We used the panel of site A and B mutants to partially map the epitopes of the hmAbs.

### The E05 epitope

Mutations K140I and RSNNS142-146GPGSG in antigenic site A eliminate the binding of E05 but other mutations in antigenic site A as well as mutations in antigenic site B have no effect (Table 2). Two escape mutants selected with E05 were isolated; both had changes at 140 (K140T and K140E). The E05 epitope therefore appears to be centered around amino acids 140–146. ELISA data of E05 binding to H3N2 viruses shows high affinity binding to Wisconsin/67/05 and Oklahoma/309/06, low affinity binding to California/7/04 and no binding to A/Beijing/89, A/Panama/99, A/Wyoming/03, A/Uruguay/716/07 or A/Perth/17/09 (Table 3). Uruguay/07 and Perth/09 viruses share a mutation K140I in HA1 (Figure 1B), and this is likely to be the reason why E05 does not bind to them. Wyoming/03 HA has a mutation N145K compared to Wisconsin/05 HA which is likely to be the reason for non-binding of E05.

The low binding of E05 to A/California/07/04 is not immediately explained. The only difference in site A between California/04 and Wisconsin/05 viruses is N122D with loss of an N-linked glycan, but this glycan site is present in Oklahoma/309/06, which binds E05 with high affinity when HA is expressed in insect cells or the virus is grown in mammalian cells. It is possible that the glycan added in mammalian or insect cells does not interfere with E05 binding but the longer complex glycan on the egg-grown California/07/04 virus blocks E05 binding. We have not been able to adapt A/Oklahoma/309/06 in chicken eggs to test this. In any case, our data suggest that the epitope of neutralizing antibody E05 is centered on the site A loop of the HA.

### The 1_C02 epitope

Antibody 1_C02 binds to A/Wisconsin/67/05 and A/Oklahoma/309/06 viruses, but not California/07/04 or Uruguay/716/07. An escape mutant virus selected by 1_C02 (EM1_C02) contains a single substitution, F159S (Figure 1B), in antigenic site B. ELISA analysis of mutants in site B shows that mutation HL156-157KS dramatically decreases the binding of 1_C02, but the adjacent mutation KFK158-160GST, that includes F159S, results in only a six fold decrease in affinity. 1_C02 binds to mutant NDQI189-192QEQT and A196V as well as to wild type. To try to understand these effects of mutations, we used SWISS-MODEL [45], [46] to model the structure of A/Oklahoma/309/06 HA and its mutants on to the crystal structure of H3 HA of A/California/04 determined recently in our laboratory. In California/04, H156 is stacked against F159 and also makes contact with T196. F159 is present in Wisconsin/05 and Oklahoma/309/06 but the change T196A may alter the interactions in this region. In the energy-minimized model of A/Oklahoma/309/06, F159 is in the same orientation as in California/07/04, but the orientation of the side chain of F159 is changed in the HL156-157KS mutant. We propose that re-orientation of F159 in B-loop in the structure of HA disrupts the binding of mAb 1_C02. Perhaps the increased flexibility of GST158-160 compared to KFK allows antibody 1_C02 to bind while it cannot when only F159 is changed, as in the escape mutant. Sequence changes that might explain why mAb 1_C02 does not bind A/California/07/04 are N188D, S193F, T196A and D225N. The only change that could explain why A/Uruguay/716/07 does not bind 1_C02 is L194P (Table 5). It seems most likely that antibody 1_C02 interacts with the face of the 156-loop that is distal to the 190-helix, and that mutations at F159 and L194 alter the conformation so that 1_C02 cannot bind although these side chains may not be in direct contact with antibody.

From these results we conclude that the assignment of antigenic sites A and B originally made on the X-31 structure apply, at least approximately, to modern H3 HAs. Crystal structures of antibody complexes are needed to fully define the E05 and 1_C02 epitopes but to date we have not obtained suitable crystals.

### Antibodies in human plasma samples after vaccination show decreased binding to mutant HA KFK158-160GST

Thirty years ago, Webster made a large panel of mAbs against the HA of A/Memphis/1/71 virus and used these to select escape mutants [11], [32]. The results led to the identification of four antigenic sites [5] with the fifth added by Skehel [6]. Although the escape mutants showed dramatic loss of binding when tested with mAbs used for their selection and other mAbs that recognize the same antigenic site, most of the variants showed no difference when tested with polyclonal mouse, rabbit or ferret hyperimmune sera. The exceptions were mutants with changes at 140 and 145 [32]. The conclusion was that site A was immunodominant in the early H3N2 viruses. We have made a detailed study of recognition of sites A and B after 2006 and 2008 vaccinations. Of 18 subjects who were given trivalent influenza vaccine in 2006–07, 72% showed antibodies against sites A, B or both and of these, 66% showed reduced avidity against site B mutants and 39% against site A mutants. Plasma of eleven subjects vaccinated in 2008–09 were tested, including six subjects who were vaccinated in both seasons. 64% showed reduction of binding to site B mutants and only 18% against site A. Most of the site A response in 2006–07 was to the K140I mutant. In 2008–09 the Uruguay vaccine component has the K140I mutation so it is not surprising that no subjects vaccinated in 2008 discriminated the K140I mutant. The KFK158-160GST mutant was recognized by 11 of the 12 site B responders in 2006 and 7 of 7 in 2008 (Table 4). There were no changes in this region of HA1 between Wisconsin/05 and Uruguay/07 viruses, but our results suggest this is a prime candidate position for antigenic drift. Indeed, in the following vaccine strains, A/Perth/16/2009 and A/Victoria/361/2011, there is a mutation of K158N. Okada et al. [47] described a panel of mAbs generated by phage-display from a single donor that included clones that bound to site C of earlier viruses, but for 1997 and 2003 viruses most clones bound site B. Ohshima et al. found antibodies made by phage display bound a wide variety of epitopes [38], showing that there are antibodies directed to the minor antigenic sites that might select mutations in sites C, D and E but to spread, these would need to be in addition to the immunodominant site B. HA of A/Perth/16/09 compared to A/Uruguay/716/07 viruses contains mutations in antigenic site B (K158N, N189K) but also sites A (S138A) and D (K173Q). Other changes in the Perth/09 vaccine strain in site A (N144K) site D I214S, site E (E62K) and site B P194L are not found in other strains co-circulating with Perth/16/09 or subsequent isolates and so seem not to be antigenically important. Our results suggest that in recent years, antigenic site B has been immunodominant over site A but it appears that the few people with dominant site A antibodies in 2006 allowed escape and spread of the Uruguay/Brisbane viruses because of the K140I mutation. In 2008 there was clear predominance of antibodies against site B in the population, predictive of the site B change K158N that was later seen in the Perth16/09 and Victoria/361/11 epidemic strains.

## Materials and Methods

### Ethics Statement

Written informed consent was obtained from all human subjects and the study was approved by the Institutional Review Boards of the University of Oklahoma Health Sciences Center and Oklahoma Medical Research Foundation.

### Viruses and cells

The viruses used in this study were PR8 reassortants A/California/07/2004 (CDC#2005712034), A/Wisconsin/67/2005-X161b, and A/Uruguay/716/2007 X-175, all obtained from CDC, and A/Oklahoma/309/2006, a Wisconsin-like H3N2 isolate. Viruses were grown in embryonated chicken eggs or in Madin-Darby canine kidney (MDCK) cells in DMEM: Ham's F12 medium (1∶1) with ITS+ (BD Biosciences) and trypsin added as previously described [48]. *Spodoptera frugiperda* Sf9 insect cells (Invitrogen, Cat. No 11496-015) were used as the host for baculovirus expressing wild type and mutant HAs. Sf9 insect cells were grown in BD BaculoGold™ TNM-FH Insect medium (BD Biosciences).

### Antibodies and selection of escape mutants

Human monoclonal antibodies E05 (D3–4) and 1_C02 (D1–7) have been described [43]. The VH and VL genes were isolated from single B cells of subjects vaccinated with the 2005–06 or 2006–07 trivalent subunit influenza vaccine, respectively and cloned into expression vectors to produce IgGs. The expressed antibodies were purified on Protein A columns. Escape mutants were selected by incubating A/Wisconsin/67/05-X161b with 1_C02 or E05 mAb and inoculating MDCK cells in a 6-well plate with varying dilutions of the antibody-virus mixture. Escape mutants that grew out were passaged three times at limiting dilution then the HA1 region was sequenced.

### Plasmid construction and mutagenesis

The HA gene of A/Oklahoma/309/06 was cloned into plasmid pCAGGS/MCS [49] as described [50]. For transient expression of wild type and mutant HA proteins, HeLa or COS cells were grown in DMEM containing 10% supplemented calf serum (HyClone), 1% glutamine, 1% sodium pyruvate and 1% PKS (penicillin, streptomycin, kanamycin sulfate). Wild type and mutant HA plasmid expression vectors were transfected into the mammalian cells using Lipofectamine™ 2000 transfection reagent (Invitrogen) according to the manufacturer's instruction. To quantify the binding of HA and mutants to human monoclonal antibodies 1_C02 and E05 [43] and antibodies in human plasma samples after vaccination we used a Baculovirus expression system. An insect cell codon-optimized HA gene of A/Oklahoma/309/06 was synthesized by GeneArt (Regensburg, Germany). The construct included the N-terminal gp67 secretion signal peptide, then residues 1–512 of HA, the trimerization foldon sequence [40], thrombin cleavage site and His6-tag. Wild type and mutant HA were cloned into the pFastBac™ expression vector for expression in the Bac-to-Bac® Baculovirus expression system (Invitrogen).

To introduce mutations into antigenic sites A and B the QuickChange® Site-Directed Mutagenesis Kit (Agilent Technologies) was used. Mutagenic oligonucleotides were named according to the original amino acid(s), their position, and the residue(s) that are changed. The following mutagenic oligonucleotides were used:

Antigenic site A:

forw Bac NNES121-124ITEG: GCACCCTCGAGTTCATCACCGAGGGCTTCAACTGGACCGGTG

forw Bac N126T: ACAACGAGTCCTTCACCTGGACCGGTGTCAC

forw Bac N133D: CCGGTGTCACCCAGGACGGCACCTCCTCCTC

forw Bac TSSS135-138GSNA: GGTGTCACCCAGAACGGCGGCTCCAACGCTTGCAAGCGTCGTTCCAC

forw Bac K140I: CCTCCTCCTCTTGCATCCGTCGTTCCAACAACTC

Forw Bac RSNNS142-146GPGSG: CACCTCCTCCTCTTGCAAGCGTGGTCCCGGCAGCGGCTTCTTCTCCCGTCTGAACTGG

Antigenic site B:

forw BacHL156-157KS: GTCTGAACTGGCTGACCAAATCGAAGTTCAAGTACCCCGCTC

forw Bac KFK158-160GST: AACTGGCTGACCCACCTGGGGTCCACGTACCCCGCTCTGAACGTG

forw Bac NDQI189-192QEQT: GCACCACCCCGGCACCGACCAAGAGCAGACCTTCCTGTACGCTCAGGC

forw Bac A196V: CCAGATCTTCCTGTACGTTCAGGCTTCCGGTCGTATC

All mutations were confirmed by sequence analysis of the whole HA1 coding region.

### Expression of wild type and mutant HAs

To produce recombinant baculovirus containing wild type and mutant HAs, SF9 insect cells were transfected with the pFastBac™ HA construct using Cellfectin®II reagent (Invitrogen™) according to the manufacturer's protocol. After 72 hours the supernatant containing P1 viral stock was checked for expression of HA protein by Western blot analysis. Positive P1 viral stocks were used to generate high-titer P2 baculoviral stocks that were used for large scale (200 ml) production of HAs. HAs were purified on a nickel column and tested for correct folding and processing into HA1 and HA2 by trypsin digestion.

### Quantification of expressed wild type and mutant HAs

HAs expressed in the Baculovirus system were quantified by a Western immunoblotting assay. Protein samples were loaded after boiling in 2× loading buffer (10 M urea, 4% SDS, 2% β-mercaptoethanol, 112.5 mM Tris pH 6.8, 0.01% bromphenol blue) and run in 12% SDS-PAGE. Proteins were electroblotted to Immobilon™-P transfer membrane (Millipore) in buffer (200 mM CAPS pH 11.0, 10% v/v methanol) for 2 h. To detect HAs we used goat polyclonal anti-HA tag (YPYDVPDYA, conserved in H3 HA) antisera (Novus Biologicals®). The blots were developed with 10 ml bromochloroindolyl phosphate and nitroblue tetrazolium substrate solution (Sigma). The bands were scanned and quantitated using ImageQuant software (Molecular Dynamics) and amounts of HA were determined from a standard curve of GST-HA tag (YPYDVPDYA) fusion protein.

To measure the amount of HA that was correctly folded we used a native enzyme-linked immunosorbent assay (ELISA). HA (50 µg) expressed from SF9 cells was captured on His-Tag® Antibody Plate wells (Novagen®) and incubated overnight at 4°C. Wells were washed 3 times with PBS, then serial dilutions of human monoclonal antibodies or heat-inactivated plasma were added to the wells and incubated at room temperature for 1 hour. After washing in PBS, alkaline-phosphatase-conjugated goat anti-human polyvalent immunoglobulin (α, γ and μ-chain specific) secondary antibody (Sigma#3313) was bound for 1 hour, the wells washed free of unbound conjugate and ρ-nitrophenyl phosphate substrate (Sigma #104) added. The color was developed at room temperature for 1 hour and absorbance was read at 405 nm. Antibody affinities were calculated by nonlinear regression (Y = Bmax×X/(Kd+X)) (GraphPad Prism software,) of ELISA curves plotted from eight dilutions of antibody or human plasma and represented as the dissociation constant (Kd) calculated in terms of binding sites (half-IgG). For human plasma, this is an overall or apparent Kd. A Kd increase greater than or equal to 1.3 is considered a significant reduction of binding of antibodies in human plasma samples.

### Native ELISA to quantitate antibodies against viruses

We used turkey red cell ghosts solubilized with β-octylpyranoside to capture native virions for ELISA as previously described [51]. The turkey red blood cells were purchased from Lampire Biological Laboratories, Inc.

## Supporting Information

Table S1 Overall affinity (Kd) of binding of human antibodies to 309 HA and mutants after vaccination against A/Wisconsin/67/05 in the 2006–07 trivalent vaccine.
(PDF)

Table S2 Overall affinity (Kd) of binding of human antibodies to 309 HA and mutants after vaccination against A/Uruguay/716/07 in the 2008–09 trivalent vaccine.
(PDF)
