# Supplementary material for: Immunodominance of Antigenic Site B over Site A of Hemagglutinin of Recent H3N2 Influenza Viruses
Source: PLoS One. 2012 Jul 25;7(7):e41895. doi: 10.1371/journal.pone.0041895 (PMC3405050; doi:10.1371/journal.pone.0041895)
Supplement: Table S1 — Overall affinity (Kd) of binding of human antibodies to 309 HA and mutants after vaccination against A/Wisconsin/67/05 in the 2006–07 trivalent vaccine. (PDF) [file pone.0041895.s001.pdf]

Table S1. Overall affinity (Kd) of binding of human antibodies to 309 HA and mutants after vaccination against A/Wisconsin/67/05 in the 2006-07 trivalent vaccine.

sample	Kd, 10^{-3} µl plasma ± SD										
	309 HA	HL156-157KS	KFK158-160GST	NDQI189-192QEQT	A196V	NNES121-124ITEG	N126T	N133	TSSS135-138GSNA	K140I	RSNNS142-146GPGSG
048	11.6±0.2	9.9±0.1	11.9±0.6	10.7±0.2	10.0±4.0	8.6±2.9	9.8±5.1	10.0±3.9	12.0±1.9	13.8±3.0	9.4±2.8
x14	11.3±0.3	14.0±0.2	22.4±0.4	13.1±0.6	12.0±3.0	12.4±2.6	9.6±4.8	11.9±3.7	12.2±3.5	15.6±3.5	16.2±6
006	4.3±0.4	5.0±0.2	3.3±0.8	3.5±0.1	3.1±1.8	2.9±0.4	3.8±1.1	3.2±0.4	2.9±0.4	4.8±0.7	3.1±1.1
119	5.7±0.5	7.8±0.3	4.9±0.8	4.4±0.6	4.9±0.6	6.8±0.4	5.6±0.6	5.6±0.5	5.2±0.8	6.9±1.5	6.1±1.8
078	2.2±0.3	3.0±0.3	3.5±0.5	3.3±0.3	2.4±0.3	1.8±0.4	2.4±1.2	2.3±0.4	1.7±0.5	2.8±1.2	2.6±0.4
082	5.4±0.9	8.7±0.7	7.2±1.1	6.7±3.8	6.1±0.7	4.4±1.8	5.5±1.4	3.2±0.4	3.7±0.7	3.8±1.4	3.3±1.4
256	4.4±1.7	5.4±2.8	2.3±0.5	4.2±0.1	4.5±1.1	8.8±0.9	9.2±1.3	9.3±4.4	4.1±2.2	14.0±1.0	9.0±1.8
014	3.1±1.4	3.8±0.9	3.6±1.3	2.2±1.0	3.0±0.5	3.7±1.6	3.4±0.8	3.9±1.6	4.0±1.0	3.8±0.9	3.2±1.9
080	27.8±7.4	17.0±1.5	29.2±1.4	31.3±11.9	28.7±6.8	33.2±3.0	20.2±5.7	26.0±9.3	18.8±5.2	19.7±5.1	23.2±5.1
013	2.0±0.6	2.1±0.1	2.2±0.1	1.9±0.2	2.0±0.8	1.9±0.3	1.7±0.5	1.5±0.5	2.1±0.9	1.6±0.7	1.7±0.7
040	6.3±2.4	8.0±1.3	11.9±2.1	8.2±2.6	7.0±1.7	8.8±3.5	8.4±3.9	10.5±4.0	7.5±2.5	9.6±3.1	7.2±2.1
061	10.8±2.8	12.7±0.7	29.9±2.3	15.9±4.2	11.0±2.7	13.0±2.0	13.1±2.8	11.8±2.0	13.2±1.9	10.1±2.5	10.5±1.3
049	9.2±3.8	14.2±2.6	15.4±1.9	10.7±1.1	7.6±1.7	12.4±3.0	9.6±1.6	15.6±5.0	12.2±4.1	22.7±3.1	16.2±2.3
030	2.6±0.6	3.8±0.8	4.0±0.7	3.7±0.4	2.4±1.0	2.6±1.1	2.4±0.4	2.0±0.5	2.5±0.6	2.3±0.4	1.7±0.3
200	9.0±1.6	16.5±0.9	13.3±3.2	10.1±1.0	6.9±1.3	5.4±1.4	6.5±1.5	6.9±0.9	5.4±1.0	11.9±0.4	6.1±1.0
182	12.7±0.6	12.7±1.2	22.3±4.6	10.2±4.5	10.9±1.1	9.8±0.6	10.7±1.6	12.3±1.1	12.5±0.6	11.2±1.0	13.0±3.0
016	2.8±1.2	3.1±0.3	6.8±0.8	3.9±0.6	3.8±0.9	3.9±0.9	3.7±1.2	2.8±1.1	4.1±1.3	3.1±1.5	3.5±1.1
y14	15.3±3.9	16.0±4.5	23.3±3.3	10.4±6.5	11.0±2.8	15.1±2.3	8.8±2.1	9.1±3.7	8.1±0.7	25.2±0.9	9.7±0.8
