# Supplementary material for: Immunodominance of Antigenic Site B over Site A of Hemagglutinin of Recent H3N2 Influenza Viruses
Source: PLoS One. 2012 Jul 25;7(7):e41895. doi: 10.1371/journal.pone.0041895 (PMC3405050; doi:10.1371/journal.pone.0041895)
Supplement: Table S2 — Overall affinity (Kd) of binding of human antibodies to 309 HA and mutants after vaccination against A/Uruguay/716/07 in the 2008–09 trivalent vaccine. (PDF) [file pone.0041895.s002.pdf]

Table S2. Overall affinity (Kd) of binding of human antibodies to 309 HA and mutants after vaccination against A/Uruguay/716/07 in the 2008-09 trivalent vaccine.

sample	Kd, 10^{-3} µl plasma ± SD										
	309 HA	HL156-157KS	KFK158-160GST	NDQI189-192QEQT	A196V	NNES121-124ITEG	N126T	N133	TSSS135-138GSNA	K140I	RSNNS142-146GPGSG
022	10.4±1.4	13.7±3.2	26.6±7.0	16.8±6.0	10.9±2.9	10.0±3.3	11.8±3.6	11.2±3.0	10.0±2.7	10.9±1.8	11.7±3.1
062	12.9±3.6	15.2±4.5	12.6±2.7	9.7±3.9	11.9±1.3	10.7±3.5	11.7±3.6	12.2±2.9	10.0±2.7	14.4±1.8	14.2±3.7
040	31.0±1.6	21.0±2.4	21.7±5.0	26.3±3.8	34.0±5.9	18.7±4.2	20.8±1.9	31.3±8.4	21.3±5.7	33.2±10.5	21.7±1.1
065	16.8±3.2	11.4±2.6	20.5±7.8	19.2±1.3	17.1±6.5	25.6±4.9	25.2±6.9	27.3±6.7	18.3±4.5	21.4±5.0	28.7±8.0
057	0.8±0.1	0.8±0.01	1.7±0.5	0.8±0.3	0.7±0.09	0.9±0.1	0.9±0.01	0.8±0.1	0.7±0.1	0.9±0.1	0.5±0.01
131	5.1±2.4	7.9±3.0	15.3±2.9	5.6±2.1	5.0±3.8	8.8±4.0	6.7±1.5	5.1±2.4	5.0±1.4	5.4±1.5	7.8±3.9
030	7.3±1.3	7.0±0.5	9.1±0.9	9.0±1.5	8.6±0.8	8.1±1.3	8.4±0.9	6.5±0.8	8.2±1.5	7.6±1.3	8.0±1.7
200	4.0±1.5	8.0±0.3	9.1±0.9	6.8±1.3	5.2±2.5	4.0±0.9	4.9±1.0	4.6±0.4	5.0±1.4	4.2±1.0	3.7±1.5
182	5.2±0.9	5.3±0.7	7.2±0.2	5.3±1.1	5.1±2.1	5.9±1.2	4.7±0.8	6.1±0.7	4.6±0.9	4.9±0.7	6.4±1.9
016	3.3±0.9	4.0±0.4	7.9±0.6	3.5±0.9	2.5±0.3	2.9±0.8	3.0±1.0	3.0±0.9	2.8±0.5	3.3±0.9	2.7±0.9
y14	13.2±2.7	14.8±3.1	18.3±2.1	12.4±2.8	12.0±2.8	13.3±4.6	13.1±2.5	12.0±3.1	12.1±2.4	11.4±2.7	13.6±3.7
